# Exploring Individual and Structural Factors Associated with Employment Among Young Transgender Women of Color Using a No-Cost Transgender Legal Resource Center

**DOI:** 10.1089/trgh.2016.0034

**Published:** 2017-03-01

**Authors:** Brandon J. Hill, Kris Rosentel, Trevor Bak, Michael Silverman, Richard Crosby, Laura Salazar, Michele Kipke

**Affiliations:** ^1^ Department of Obstetrics and Gynecology, Center for Interdisciplinary Inquiry and Innovation in Sexual and Reproductive Health (Ci3), The University of Chicago, Chicago, Illinois.; ^2^ Section of Family Planning and Contraceptive Research, Department of Obstetrics and Gynecology, University of Chicago, Chicago, Illinois.; ^3^ The Kinsey Institute for Research in Sex, Gender, and Reproduction, Indiana University, Bloomington, Indiana.; ^4^ School of Social Service Administration, University of Chicago, Chicago, Illinois.; ^5^ Transgender Legal Defense & Education Fund, Inc., New York, New York.; ^6^ College of Public Health, University of Kentucky, Lexington, Kentucky.; ^7^ School of Public Health, Georgia State University, Atlanta, Georgia.; ^8^ Department of Pediatrics, Children's Hospital Los Angeles, Los Angeles, California.

**Keywords:** employment, structural intervention, transgender, transgender health, transgender rights

## Abstract

**Purpose:** The purpose of this study was to explore individual and structural factors associated with employment among young transgender women (TW) of color.

**Methods:** Sixty-five trans women of color were recruited from the Transgender Legal Defense and Education Fund to complete a 30-min interviewer-assisted survey assessing sociodemographics, housing, workplace discrimination, job-seeking self-efficacy, self-esteem, perceived public passability, and transactional sex work.

**Results:** Logistic regression models revealed that stable housing (structural factor) and job-seeking self-efficacy (individual factor) were significantly associated with currently being employed.

**Conclusion:** Our findings underscore the need for multilevel approaches to assist TW of color gain employment.

## Background

Transgender women (TW; trans women) face immense social and economic marginalization and experience a host of health disparities, particularly trans women of color. Although prevalence estimates are limited, a meta-analysis of 29 studies in the United States estimated that roughly 28% of trans women participants had tested positive for HIV, with 12% self-reporting a known HIV-positive status.^1^ However, such stark disparities in HIV infection cannot be attributed to individual level behavior alone, but are situated in larger social and structural contexts that proximate young trans women of color to increased HIV exposure, including social and economic marginalization.^2–9^

The interaction between the individual and social and structural factors associated with health has been described by syndemic theory, or the synergistic interplay between co-occurring health problems and the social environment of a specific group or community.^10^ One key premise of syndemic theory is that adverse social conditions determine the concentration of poor health outcomes.^10^ Thus, using a syndemic framework, previous research has demonstrated that health problems among trans women may also be determined by adverse social and structural factors.^11–17^ Indeed, despite increased legal protections and rights for lesbian, gay, and bisexual people, few laws specifically protect transgender people from discrimination, including employment and housing discrimination.^9,18,19^ As a result, a fair majority of young trans women of color live in poverty, experiencing high rates of unemployment, homelessness, and limited access to healthcare.^16,20–22^ In particular, the 2011 National Transgender Discrimination Survey (NTDS) reports that roughly 28% of black and 18% of Latino/a transgender respondents were unemployed, four times, and twice, the rate of the general population, respectively.^5^ Faced with few economic options, studies suggest that young trans women may migrate to nontraditional economies or “street careers” as a means of survival.^18,21,23,24^ In a study of young trans women of color in Los Angeles and Chicago, 76% of the 151 participants aged 15–24 reported engaging in transactional sex work, with 35% having engaged in sex work in the past 3 months.^24^ In addition, unemployment has been associated with several other poor health outcomes among trans women, including mental health issues and substance use.^25–27^

More recently, researchers have begun to consider the potential impact of the social environment on the health of young trans women, emphasizing the need to attend to structural and policy level factors.^11–14,16,17,21^ In a recent review of the behavioral interventions to prevent HIV transmission and acquisition for trans women, Garofalo et al. articulated, “given the profound socioeconomic instability—which far-too-often is a social reality for TW [transgender women]—and its influence on the initiation and engagement in high-risk behaviors such as transactional sex, the development of structural approaches such as employment and vocational interventions and initiatives should be strongly considered and prioritized because they offer the greatest promise at holistically improving the lives of TW, including holding promise for the reduction of HIV transmission.”^17^ However, although there are several studies on the social and health consequences of unemployment among trans women, little is known about what factors may facilitate transgender employment.

The purpose of this study was to explore the individual and structural level factors associated with current employment among a sample of 65 young trans women of color enrolled in a no-cost legal name change clinic. More specifically, this study considers how specific structural factors, such as education, housing, workplace discrimination, incarceration, and legal name status on identification documents, and individual factors, such as job-seeking self-efficacy, perceived confidence passing in public, self-esteem, HIV status, and engagement with transactional sex work, relate with employment. Given the extant literature on transgender employment, we hypothesize that key structural factors (e.g., education, housing, workplace discrimination, and legal name change status on identification documents)^2,4,5,7–9^ will be associated with the current employment status of young trans women of color. Furthermore, given the research literature on employment among marginalized populations, we hypothesize that individual factors (e.g., job-seeking self-efficacy, confidence passing in public, self-esteem, HIV-status, transactional sex work)^7–9,18,19,24,28–30^ will also be associated with the employment status of young trans women of color.

## Methods

### Participants and procedures

Data were collected as part of a larger university and non-for-profit partnership study exploring the potential effect of legal name change on the economic and health status of TW of color using the Transgender Legal Defense and Education Fund (TLDEF) Name Change Project (NCP), a program that provides no-cost legal name change legal services to low-income transgender people. Baseline data were used for the present study. All participants were recruited through the TLDEF-NCP. Eligible participants included those who identified as a transgender woman or trans woman; aged 18–35 years; self-identifying as a person of color or black/African American, Hispanic/Latina, and/or Asian/Pacific Islander. Data were collected via a 30-min interviewer-assisted telephone survey. Survey interviews were conducted from March 2015 to October 2015. Survey items assessed sociodemographic characteristics, education level completed, incarceration history, housing stability (past year), work discrimination, job seeking self-efficacy, self-esteem, and self-perceived confidence “passing” in public. All participants gave oral informed consent and received a $20 gift card for participating in the study. Written informed consent was waived for the protection of participant confidentiality. All procedures were approved by the university's Institutional Review Board.

### Measures

#### Sociodemographic characteristics

Participants reported their age, highest education level attained (less than high school, currently in high school, high school graduate, some college, currently in college, college graduate, more than college graduate), ever been incarcerated (yes/no), current employment status (yes/no), legal name change status (prename change/postname change), and HIV status (positive, negative, do not know).

#### Housing stability

Housing stability over the past 12 months was assessed using the question: “What are the different types of places that you lived in the past year?” Response options included the following: apartment or house they paid for, someone else's house, motel, car, halfway house, sober living, rehabilitation home, and somewhere else. The response option “an apartment or house they paid for” was coded as “stably housed.”

#### Work-related discrimination based on transgender identity

One item assessed work discrimination, “In the past 6 months, have you experienced discrimination at work (your place of employment) as a result of being transgender?” Response options yes/no.

#### Job-seeking self-efficacy

Job-seeking self-efficacy was measured using the Job-Seeking Self-Efficacy Scale (JSS), which consists of seven items asking about the respondent's sense of self-efficacy to complete various tasks associated with applying for jobs.^31^ Respondents indicated yes=1 or no=0 to each item; items were summed and then divided by the total completed to produce an overall mean JSS score, with higher scores indicating higher job-seeking self-efficacy.^31^

#### Self-esteem

The Rosenberg Self-Esteem Scale, consisting of 10 items, to which responses ranged on a Likert scale from 1=Not at all supportive to 4=Very supportive. Items were recorded so that higher scores represented more positive feelings of self-worth. The mean score across all items were used as the overall score.^32^

#### Self-perceived confidence “passing” as their gender identity

One item, “Do you feel you confidently ‘pass’ as a woman in most public places? (yes/no)” was used to assess self-perception of passability in public.

#### Transactional sex work

One item, “In the past 6 months, have you had anal sex with a male in exchange for money or drugs?” was used to assess recent experience with sex work associated with HIV risk.^33–35^ Participants indicated yes or no.

### Data analysis

Descriptive statistics analyzed proportions and central tendencies for all demographic characteristics as well as individual- and structural-level variables. A logistic regression model, including sociodemographic characteristics, housing stability, work discrimination, job-related self-efficacy, self-esteem, and self-perceived possibility as independent variables, was built to examine associations between individual- and structural-level variables and employment status. All data were analyzed in SPSS 22.0 (Chicago, IL).

## Results

### Sample characteristics

Table 1 details the individual- and structural-level characteristics of the sample. Approximately 43.1% of young trans women of color were high school graduates, and 40.0% had some amount of college education. A total of 43.1% reported having stable housing (house or apartment they paid for) over the past year, 29.7% reported being employed, and 30.2% reported having experienced work-related discrimination in the past 6 months. One-fifth (20.0%) self-reported being HIV-positive and 26.6% reported having had sex for drugs or money (i.e., transactional sex work) in the past 6 months. The majority of participants (81.0%) reported self-perception as passing in public as their gender identity. Forty-three percent reported having changed their legal name.

**Table 1.** **Sociodemographic, Structural- and Individual-Variables (*N*=65)**

Variable	*n*	%^a^
Sociodemographics		
Age, mean (SD)	26.1 (4.74)	
Black/African American	34	52.3
Hispanic/Latina	30	46.2
Asian/Pacific Islander	5	7.7
Currently employed	19	29.7
Structural-level variables		
Education		
Less than high school/currently in high school	11	16.9
High school graduate	28	43.1
Any college	26	40.0
Stable housing=apartment or house you own or rent (past year)	28	43.1
Work-related discrimination due to transgender identity (past 6 months)		
Have experienced discrimination at work for transgender identity	19	30.2
Ever been incarcerated	19	29.2
Have changed legal name	28	43.1
Individual-level variables		
Confident in “pass” in public	51	81.0
HIV-positive status	13	20.0
Transactional sex work (past 6 months)	17	26.6
Job-seeking self-efficacy score,^b^ mean (SD)	5.5 (1.33)	
Self-esteem score, mean (SD)	2.69 (0.27)	

^a^ Valid percentages.

^b^ Missing three responses.

SD, standard deviation.

### Associations between individual- and structural-level variables and employment status

As seen in Table 2, stepwise logistic regression models suggest that two main individual and structural level variables were significantly associated with current employment status. In particular, young trans women of color who reported having had stable housing in the past year (structural level variable) had a nearly six times greater odds of being employed (odds ratio [OR]=5.64, 95% confidence interval [CI]=1.20–26.46) (Model 3). In addition, every one unit increase in job self-efficacy (individual level variable) was associated with almost a two times greater odds of being employed (OR=2.90, 95% CI=1.18–7.07) (Model 3). In addition, in the full model having changed your legal name on identification documents was associated with being employed (OR=3.82, 95% CI=0.77–18.96, *p*<0.10), but this finding did not reach *p*<0.05 significance. Although roughly over one-quarter of the participants reported transactional sex in the past 6 months, sex work was not associated with employment. There were no significant associations for any other individual or structural level variables in any of the models.

**Table 2.** **Structural and Individual Factors Associated with Current Employment Status Among Transgender Women of Color (*N*=65)**

Variable	Model 1: structural factors, OR (95% CI)	Model 2: individual factors, OR (95% CI)	Model 3: full model, OR (95% CI)
Education	1.26 (0.80–1.96)	—	1.31 (0.80–2.14)
Stable housing (past year)	5.11 (1.32–19.76)^*^	—	5.64 (1.20–26.46)^*^
Work-related discrimination due to transgender identity (past 6 months)	0.34 (0.08–1.37)	—	2.52 (0.44–14.35)
Ever been incarcerated	1.29 (0.26–6.22)	—	2.08 (0.31–14.00)
Legal name change status	2.41 (0.66–8.86)	—	3.82 (0.77–18.96)^**^
Job-seeking self-efficacy score	—	2.47 (1.20–5.11)^*^	2.90 (1.18–7.07)^*^
Confident in “passing” in public	—	2.56 (0.46–14.35)	1.40 (0.18–10.80)
Self-esteem	—	1.64 (0.15–17.57)	0.42 (0.14–12.62)
HIV-status	—	0.63 (0.13–3.03)	0.21 (0.03–1.76)
Transactional sex work (past 6 months)	—	1.18 (0.30–4.54)	1.45 (0.29–7.29)

^*^ *p*<0.05, ^**^p<0.10.

CI, confidence interval; OR, odds ratio.

## Implications

To our knowledge, this is one of the first studies to explore individual and structural level factors associated with current employment status among a sample of young trans women of color. Our findings underscore two critical pathways to potentially increasing employment and addressing subsequent socioeconomic marginalization, which has been associated with negative health outcomes, including disproportionate rates of HIV infection among young trans women of color. Fortunately, both pathways are amenable to intervention. First, at a structural level, assisting displaced and/or unstably housed young trans women appears to be critical for employment and may also address a host of poor health outcomes associated with unstable housing, including increased HIV risk.^9,36,37^ Although, several housing assistance programs exist for people living with HIV, few resources are available specifically for transgender people who are HIV-negative.^27^ Indeed, nearly one in three (29%) of transgender people in the NTDS reported being turned away from a shelter due to their transgender identity.^5^ Housing may be particularly important as young women transition and may experience bouts of familial and social rejection, which leave them few housing options.^9,36,37^ Unfortunately, such rejection is met with limited legal protection against housing discrimination. In the United States, 19% transgender people have been refused a home or apartment and 11% have been evicted because of their transgender identity.^5^ Thus, policy-level changes and antidiscrimination housing laws are needed to address the constellation of factors that displace transgender people and affect their ability to gain employment.

Second, building job-seeking self-efficacy through employment and vocational training may have a direct effect on the employment status of young TW. These may be particularly advantageous as they circumvent the migration of young TW to street economies, such as transactional sex work, which greatly increases young women's risk for HIV/STI acquisition and transmission.^23,24,38^ Developing such programs and interventions are imperative and should be prioritized as they are likely to bend the curve in HIV infection rates among young trans women of color. Furthermore, creating additional employer training interventions that focus on transgender inclusivity in the workplace and creating an equitable and diverse work environment are also needed. Here again, policy level changes in workplace discrimination laws based on transgender identity may improve rates of transgender people's employment.^5^

Although our findings are promising, it is important to consider this study's limitations. First, the sample comprised trans women of color using a no-cost legal name change clinic and may not be generalizable to all trans women of color, or white trans women. Second, the data are cross-sectional, and causal inferences cannot be surmised on the relationships between transgender employment and the structural and individual level factors. Finally, our models include only a few structural and individual level factors that may influence transgender employment and more research is needed to better understand the constellation of barriers and facilitators to increasing employment access for transgender people and addressing socioeconomic stability. Factors often associated with employment, such as incarceration or HIV status, were not associated with employment in this sample. This may be a result of limited statistical power, given that only 29.2% of our 65 participants had been incarcerated and only 20.0% were living with HIV. Nevertheless, these exploratory findings emphasize the need to develop structural level interventions that target employment as an important determinant of transgender health.

## Abbreviations Used

CI: confidence interval
JSS: job-seeking self-efficacy
NCP: name change project
NTDS: national transgender discrimination survey
OR: odds ratio
STI: sexually transmitted infection
TLDEF: Transgender Legal Defense and Education Fund
TW: transgender women
